# Hypoxia induced exosomal Circ-ZNF609 promotes pre-metastatic niche formation and cancer progression via miR-150-5p/VEGFA and HuR/ZO-1 axes in esophageal squamous cell carcinoma

**DOI:** 10.1038/s41420-024-01905-8

**Published:** 2024-03-12

**Authors:** Yu Mao, Jiahao Wang, Yimin Wang, Zhanzhao Fu, Lixin Dong, Jia Liu

**Affiliations:** 1 Department of Oncology, First Hospital of Qinhuangdao, Qinhuangdao, Hebei China; 2 Key Laboratory of Research on Molecular Mechanism of Gastrointestinal Tumors in Qinhuangdao, Qinhuangdao, Hebei China; 3 Department of General Surgery, First Hospital of Qinhuangdao, Hebei Medical University, Qinhuangdao, China; 4 Department of Medical Oncology, Jerome Lipper Multiple Myeloma Center, Dana Farber Cancer Institute, Boston, MA USA

**Keywords:** Cancer, Cell biology

## Abstract

Exosomes derived from cancer are regarded as significant mediators of cancer-host crosstalk. Hypoxia, on the other hand, is one of the essential characteristics of solid tumors. This research set out to discover how circulating exosomes from hypoxic esophageal squamous cell carcinoma (ESCC) contribute to the formation of metastatic niches and distant metastasis. First, we noticed that human umbilical vein endothelial cells (HUVECs) had their tight connections disrupted and the expression of proteins involved in angiogenesis boosted by ESCC hypoxic exosomes. Hypoxia significantly induced Circ-ZNF609 expression in exosomes from ESCC, which was then internalized by HUVECs, as determined by circular RNA screening. High Circ-ZNF609 expression in HUVECs facilitated angiogenesis and vascular permeability, thereby promoting pre-metastatic niche formation, and enhancing distant metastasis in vitro and in vivo. Exosomal Circ-ZNF609 activated vascular endothelial growth factor A (VEGFA) mechanistically by sponging miR-150-5p. Exosomal Circ-ZNF609 also interacted with HuR and inhibited HuR binding to ZO-1, Claudin-1, and Occludin mRNAs, thereby reducing their translation. Collectively, our findings identified an essential function for exosomal Circ-ZNF609 from ESCC cells, suggesting the potential therapeutic value of exosomes for ESCC patients.

## Introduction

As a major hazard to human health, esophageal cancer ranks the sixth primary cause of cancer-related death and the eighth most prevalent cancer type worldwide [1]. On the basis of its pathological characteristics, esophageal cancer can be categorized as either esophageal adenocarcinoma or esophageal squamous cell carcinoma (ESCC) [2]. According to the epidemiology, ESCC, which originates from esophageal epithelial cells, is the predominant histologic type in Asian nations such as China and Japan [3]. Despite recent diagnostic and therapeutic advancements, the prognosis for esophageal cancer remains dismal [4]. The primary cause of treatment failure and poor prognosis in esophageal cancer is attributed to its prominent tendency for metastasis [5].

Currently, there is significant attention directed towards comprehending the molecular mechanisms underlying metastasis, with the aim of devising effective therapeutic interventions. As per the “seed and soil” theory proposed by Paget in 1889, neoplastic cells have the ability to exit the primary tumor site and propagate via circulation to distant organs where they can establish within a compatible microenvironment, ultimately resulting in the development of distant metastases [6]. This process involves the crucial participation of initial tumor microenvironment adaptation and pre-metastatic niche formation in the dissemination of tumor cells and their subsequent distant engraftment [7–9]. Pre-metastatic niche formation comprises various processes such as angiogenesis, vascular permeability, immune suppression, and stroma reprogramming, as previously documented in literature [10–12]. The present study centers on the function of exosomes derived from ESCC to assist pre-metastatic niches formation and enhance distant metastasis.

Exosomes derived from tumor cells are small extracellular vesicles that typically range from 30 to 100 nm in size and contain proteins, lipids, and nucleic acids. These exosomes are believed to play a role in regulating the tumor microenvironment [13, 14]. Our study has provided evidence that exosomes derived from ESCC promote angiogenesis and metastasis, as previously reported [15]. In this study, we conducted additional research to determine if exosomes derived from ESCC can promote metastasis by disrupting vascular endothelial barriers and facilitating angiogenesis, ultimately leading to the enhancement of distant pre-metastatic niche formation. Furthermore, an investigation was conducted into the involvement of hypoxia and Circ-ZNF609 in this phenomenon.

## Results

### Characterization and internalization of exosomes

**Fig. 1:**
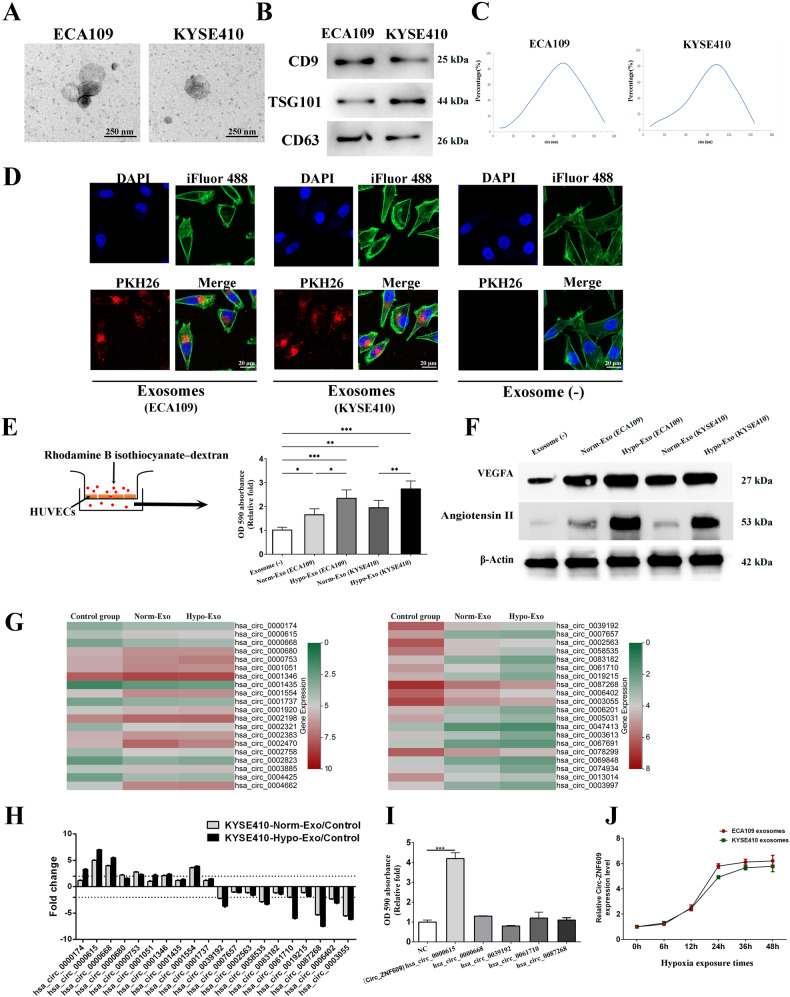
Exosomes from ESCC weaken the tight junctions of HUVECs. **A** Transmission electron micrographs of extracellular vesicles derived from ECA109 and KYSE410. **B** The expression level of CD9, TSG101 and HSP70 (exosome specific markers) in isolated extracellular vehicles. **C** The nanoparticle concentration and size distribution of the extracellular vehicles derived from ECA109 and KYSE410. **D** Internalization of exosomes from ECA109 and KYSE410 by HUVECs. **E** HUVECs were treated with exosomes isolated from hypoxic cultured- ESCC cells (Hypo-Exo) or normoxic cultured- ESCC cells (Norm-Exo) respectively. The traverse of rhodamine labeled dextran probes through HUVECs monolayers were quantified through evaluating absorbance at 590 nm. **F** The expression level of angiogenesis related protein including VEGFA and Angiotensin II. HUVECs were incubated with Hypo-Exo from KYSE410, Norm-Exo from KYSE410 or without exosomes (control group) and then their circular RNA expressions profile was analyzed. **G** Heat map of the top 20 circular RN As which experienced an up-regulation (left) or down-regulation (right) in both Norm-Exo group and Hypo-Exo group. **H** Validation of the potentially dys-regulated circular RNAs in HUVECs internalized with exosomes. **I** HUVECs were transfected with circular RNA overexpression plasmid and an in vitro permeability assay was applied to determine the barrier function of endothelial cells. **J** The expression level of Circ-ZNF609 in ESCC cells exposed to indicate time in hypoxia. Data are expressed as mean ± SD. (*p < 0.05; **p < 0.01; ***p < 0.001).

The ECA109 and KYSE410 cell lines were selected as models for determining the impact of exosomes secreted by ESCC on vascular cells. Transmission electron microscopy utilization revealed that exosomes gained from the conditioned medium of ECA109 and KYSE410 displayed a characteristic morphology of particles that were encapsulated by a membrane, as depicted in Fig. 1A. The results of the Western blot analysis indicated that the extracellular vesicles that were purified exhibited positivity for markers that are specific to exosomes, which included CD9, TSG101, and CD63 (as depicted in Fig. 1B). Furthermore, an analysis using nanoparticle tracking was conducted on extracellular vesicles that had undergone purification, revealing a size spectrum spanning from 20 to 200 nm (Fig. 1C).

Following this, an investigation was conducted to determine if exosomes derived from ESCC could be internalized by HUVECs. Initially, PKH-26 was employed to stain exosomes, which were later mixed into the culture medium of HUVECs. Following a 24-h incubation period, HUVECs were subjected to staining with iFluor 488 for Phalloidin to visualize actin filaments and DAPI for nuclear staining. The findings indicate that HUVECs exhibited a notable uptake efficacy of exosomes, as evidenced by the presence of PKH-26 fluorescence in over 90% of the cells’ cytoplasm. The findings of this study validate that the exosomes obtained from ESCC were of adequate purity for internalization by HUVECs and for conducting subsequent experiments, as demonstrated in Fig. 1D.

### Exosomes derived from ESCC disrupt the barrier role of endothelial monolayers and facilitate the process of angiogenesis

At first, HUVECs were cultured with exosomes isolated from either hypoxic cultured ESCC cells (referred to as Hypo-Exo) or normoxic cultured ESCC cells (referred to as Norm-Exo). To assess the impact of exosomes derived ESCC on the regulation of endothelial cell barrier function, an in vitro permeability assay was conducted. To achieve this, we monitored the diffusion of rhodamine-labeled dextran across the monolayers of HUVECs. Based on the analysis of absorbance at a wavelength of 590 nm, it was observed that the administration of both Norm-Exo and Hypo-Exo resulted in rhodamine-labeled dextran migration from the upper to the lower well. Furthermore, it can be observed that Hypo-Exo exhibited a superior capacity in promoting vascular permeability. (Fig. 1E)

We also conducted the assessment of the angiogenic properties of exosomes derived from ESCC. This was accomplished through an analysis of the expression levels of Vascular Endothelial Growth Factor A (VEGFA) and Angiotensin II. The findings indicate that exosomes derived from ESCC, particularly Hypo-Exo, elicited a marked upregulation of angiogenesis-associated proteins such as VEGFA and Angiotensin II. (Fig. 1F)

### Identification of significant circRNAs via Microarray analysis in HUVECs treated with exosomes from ESCC

The impact of Norm-Exo and Hypo-Exo on vascular endothelial barriers was examined, followed by a microarray analysis to uncover the underlying mechanism. The exploration of circular RNA expression profiles was conducted in a prior study [15]. In the present research, we conducted a re-analysis of the circular RNAs that were differentially expressed in HUVECs that had internalized Norm-Exo or Hypo-Exo. By conducting a comparative analysis of the circular RNA profile between the Norm-Exo and Hypo-Exo groups, we were able to identify circular RNA molecules that underwent either up- or down-regulation in both groups.

The analysis identified 692 down-regulated circular RNAs and 86 up-regulated circular RNAs at the intersection. Figure 1G displays heat maps illustrating the expression levels of the top 20 up-regulated or down-regulated circular RNAs. To confirm the efficacy of the circular RNA microarray, the PCR technique was employed to determine the expression level of the top 10 circular RNA candidates in HUVECs that were cultured with exosomes from KYSE410. Two criteria were utilized in the significant differentially expressed circular RNA screening process. The first criterion required genes to be significantly up- or down-regulated in both the Norm-Exo and Hypo-Exo groups, with a fold change above 2. The second criterion required genes to exhibit a higher level of dysregulated expression in the Hypo-Exo group compared to the Norm-Exo group.

The study identified five circular RNAs, namely hsa_circ_0000615, hsa_circ_0000668, hsa_circ_0039192, hsa_circ_0061710, and hsa_circ_0087268, as potential candidate genes (Fig. 1H). Following this, HUVECs were transfected with plasmids that overexpressed 5 circular RNAs that were identified as candidates. The up-regulation of hsa_circ_0000615 (Circ-ZNF609) was found to enhance the permeability of rhodamine labeled dextran probes through HUVECs monolayers, as demonstrated by the in vitro permeability assay (Fig. 1I). There was no statistically significant variation detected in the remaining four circular RNAs. Subsequently, it was observed that the expression levels of Circ-ZNF609 rose in exosomes under hypoxic conditions in a time-dependent manner, as depicted in Fig. 1J.

### Characterization of Circ-ZNF609 and function exploration in HUVECs

**Fig. 2:**
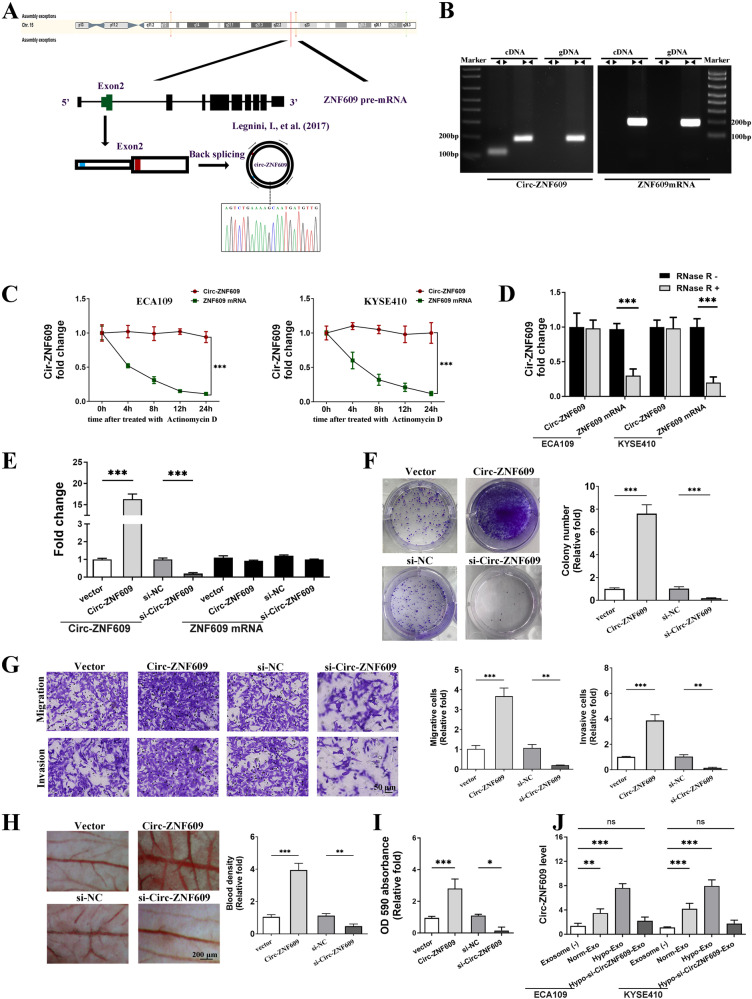
Circ-ZNF609 validation and characterization in HUVECs. **A** Schematic representation of the formation of circ-ZNF609. The 5′ and 3′ UTRs are indicated by smaller boxes, while the coding region (CDS) is represented by larger boxes. Start and stop codons are shown in red and blue, respectively. The back splice junction sequence of Circ-ZNF609 was verified by Sanger sequencing. **B** qRT-PCR products with divergent primers showing circularization of Circ-ZNF609. The divergent primers detected circular RNAs in cDNA but not in gDNA. **C** The levels of Circ-ZNF609 and ZNF609 mRNA expression in HUVECs treated with Actinomycin D (2.5 μg/ml) at the indicated time points were detected by qRT-PCR. **D** qRT-PCR analysis of Circ-ZNF609 and ZNF609 mRNA after treatment with or without RNase R in HUVECs. **E** The expression level of Circ-ZNF609 and ZNF609 mRNA in HUVECs transfected with overexpression plasmid and si-Circ-ZNF609. **F** Colony formation assays were applied to determine the proliferation of HUVECs. **G** Transwell assays were used to investigate the migratory and invasive abilities of HUVECs. **H** Representative images of chicken chorioallantoic membrane photographed on plastic dished after resection from eggs. **I** The barrier function of HUVECs monolayer was determined by in vitro Rhodamine B-dextran inter-cellular permeability assay. **J** RT-PCR results of Circ-ZNF609 level in HUVECs treated with exosomes isolated from hypoxic cultured ESCC cells (Hypo-Exo), normoxic cultured- ESCC cells (Norm-Exo) or Hypo-Exo loaded with Circ-ZNF609 siRNA (Hypo-si-CircZNF609-Exo). (*p < 0.05; **p < 0.01; ***p < 0.001).

The circular RNA ZNF609, also known as Circ-ZNF609 and identified by the circBase ID hsa_circ_0000615 (alias hsa_circ_000193), is situated on chromosome 15. This RNA molecule is generated through the circularization of the second exon of the ZNF609 gene, as illustrated in Fig. 2A. The Sanger sequencing technique was employed to confirm the back-splice site of Circ-ZNF609, as depicted in Fig. 2A. PCR analysis was able to detect the divergent primers for Circ-ZNF609 from complementary DNA (cDNA) exclusively, while genomic DNA (gDNA) did not yield any amplification. In contrast, it was observed that the divergent primers designed for linear ZNF609 mRNA as a control were not effectively amplified in either genomic DNA or complementary DNA, as depicted in Fig. 2B. In addition, the examination of Circ-ZNF609 stability was conducted through the application of Actinomycin D treatment, a transcriptional inhibitor of RNA synthesis. The findings indicate that Circ-ZNF609 exhibited a substantially longer half-life in comparison to linear ZNF609 mRNA, as demonstrated in Fig. 2C. It was observed that the resistance of Circ-ZNF609 to exonuclease ribonuclease R (RNase R) treatment was higher compared to the linear ZNF609 mRNA, which was easily degraded upon treatment (Fig. 2D). These findings indicate that Circ-ZNF609 is a highly prevalent and consistently expressed circular RNA in HUVECs.

Gain and loss-of-function tests in HUVECs were carried out to investigate Circ-ZNF609’s influence on angiogenesis. The circular RNA over-expression vector can stably generate circ-ZNF609 RNA at high levels, as previously shown [16]. In the first stage, we synthesized siRNA targeting the back-spliced junction of Circ-ZNF609. The results demonstrated that si-Circ-ZNF609 caused a substantial decrease in its RNA level. Additionally, by using over-expression vectors, Circ-ZNF609 was ectopically up-regulated. There was no tested change in ZNF609 mRNA (Fig. 2E). The proliferation, viability, and angiogenesis of HUVECs were assessed using the colony formation, transwell, and chicken chorioallantoic membrane (CAM) assays. According to the findings, Circ-ZNF609 overexpression dramatically increases the ability of cells to proliferate, migrate, and invade (Fig. 2F, G). With Cir-ZNF609 overexpression, the chicken chorioallantoic membrane had more microvascular, but Circ-ZNF609 knockdown had the reverse effect (Fig. 2H). Additionally, the leakage of rhodamine-B dextran across HUVEC monolayers was made more intense by Circ-ZNF609 up-regulation (Fig. 2I). These results suggested that Circ-ZNF609 enhances permeability and angiogenesis in HUVECs.

### Hypoxia-induced exosomal Circ-ZNF609 from ESCC promotes pre-metastatic niche formation via facilitating angiogenesis and increasing vascular permeability

Next, we aimed to clarify the function of exosomal Circ-ZNF609. To achieve this, exosomes with varying levels of Circ-ZNF609 were extracted from ECA109 or KYSE410 cells maintained under normoxic or hypoxic conditions. These exosomes were then co-cultured with HUVECs, resulting in four distinct experimental groups: a control group without exosomes, a normoxic exosome group (Norm-Exo), a hypoxic exosome group (Hypo-Exo), and a hypoxic exosome group packed with si-Circ-ZNF609 (Hypo-si-Circ-ZNF609-Exo). The confirmation of the quantity of Circ-ZNF609 in HUVECs from each group was demonstrated through Fig. 2J.

**Fig. 3:**
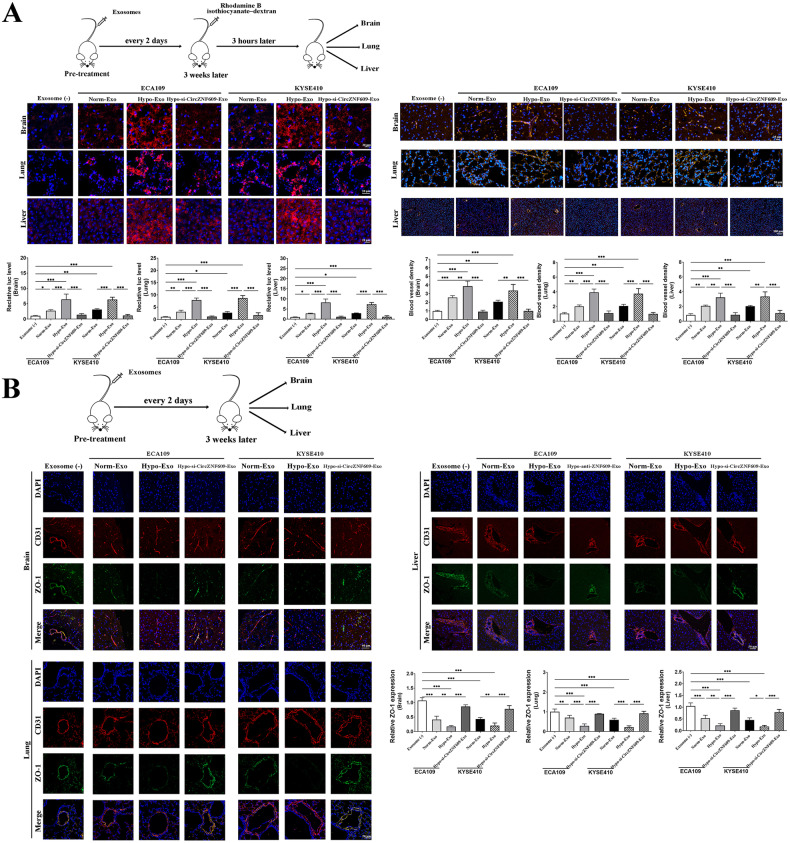
Hypoxia-induced exosomal Circ-ZNF609 enhances vascular permeability and angiogenesis in nude mice. **A** Balb/c nude mice were pretreated with hypoxic atmosphere (Hypo-Exo), normoxic atmosphere (Norm-Exo) or PBS through the tail veins exosomes every 2 days for three weeks to mimic continuous and systemic stimulation by exosomes from ESCC cells. After exosomes education, mice were sacrificed and brain, lung and liver were isolated for immunofluorescence. Rhodamine-dextran was administrated intravenously 3 h before sacrifice. Tissues were isolated to evaluate vascular density. **B** Samples were stained with CD31 and ZO-1 to show vascular permeability. (*p < 0.05; **p < 0.01; ***p < 0.001).

To assess the potential participation of exosomeal circ-ZNF609 derived from ESCCs in the formation of pre-metastatic niches and vascular permeability, we administered Rhodamine B isothiocyanate-dextran after exosome education. The organs of interest, including the liver, lung, and brain, were isolated and evaluated for their respective levels of vascular permeability. The assessment of vascular permeability was conducted through observation of the presence of intracellular rhodamine-dextran (red) after injection. The inspection of vascular density was conducted through the utilization of CD31 staining. The results depicted in Fig. 3A indicate that both Norm-Exo and Hypo-Exo facilitated the extravasation of rhodamine-dextran from the vascular system into the surrounding tissues, concomitant with the process of angiogenesis. Furthermore, the group that received Hypo-Exo administration exhibited a statistically significant increase in the extent of rhodamine-dextran penetration and blood vessel density in organs such as the liver, lung, and brain. The study uncovered a significant reduction in vascular permeability and blood vessel density in the brain, liver, and lung of mice that were administered with Hypo-si-CircZNF609-Exo.

Furthermore, exosomes that were isolated from ESCC cells were administered via the tail vein of nude mice (as depicted in Fig. 3B). Following a three-week period, we observed the expression of ZO-1 in CD31^+^ vascular endothelial cells of organs that are commonly associated with ESCC metastases, such as the brain, lung, and liver, among various groups. This was done to assess the impact of exosomes on the modulation of tight junctions in endothelial cells and vascular integrity, as depicted in Fig. 3B. ZO-1 expression was reduced in the brain, liver, and lungs after Norm-Exo treatment, in comparison to the control group. The administration of Hypo-Exo demonstrated a substantial decrease in the expression of ZO-1 in the endothelial cells of cerebral, pulmonary, and hepatic vessels. In addition, the suppression of Circ-ZNF609 within exosomes derived from ESCC counteracted the impact of hypoxic exosomes on the vascular integrity of remote organs, thereby facilitating the initiation of pre-metastatic niche establishment.

### Exosomal Circ-ZNF609 promotes ESCC metastasis and cancer progression

**Fig. 4:**
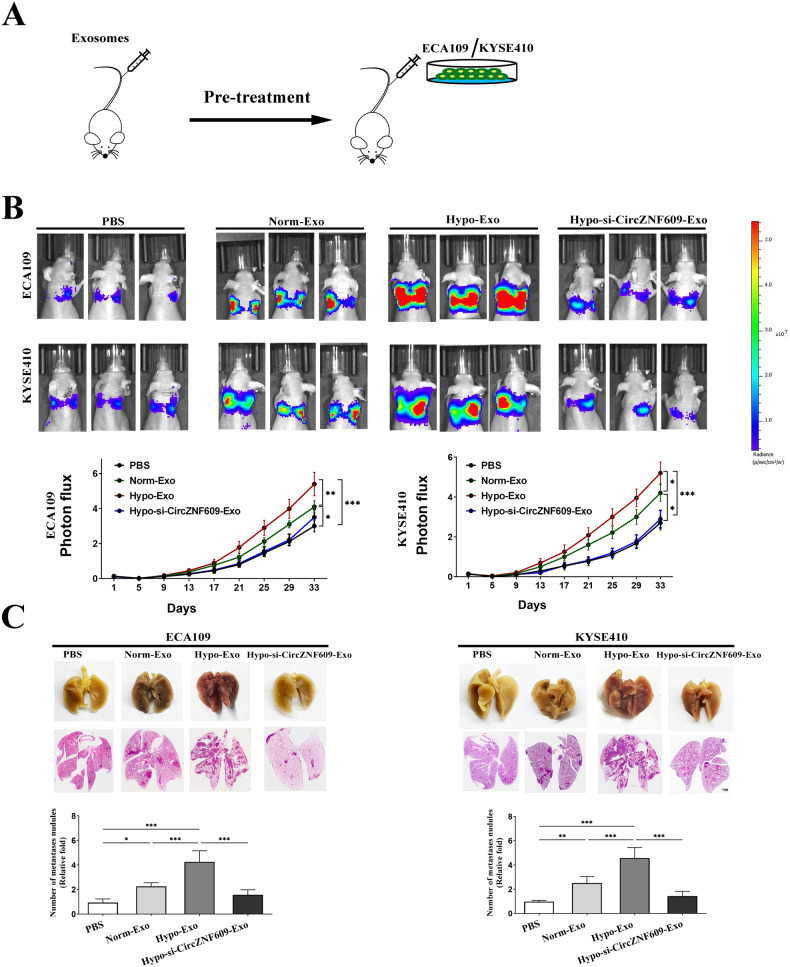
Hypoxia-induced exosomal Circ-ZNF609 primes pre-metastatic niche formation and facilitates cancer metastasis in vivo. **A** Balb/c nude mice were pretreated with hypoxic atmosphere (Hypo-Exo), normoxic atmosphere Norm-Exo or PBS through the tail veins. After exosomes education, luciferase labeled ESCC cells were injected into nude mice. **B** Photon flux curves and representative pictures of luciferase were captured on day 25. **C** After 33 days, nude mice were sacrificed, lungs were fixed and stained with H&E. Data are expressed as mean ± SD. Differences among/between groups were analyzed using one-way ANOVA followed by Holm-Sidak’s multiple comparison tests or unpaired t-test. (*p < 0.05; **p < 0.01; ***p < 0.001).

We investigated further here whether circulating exosomes from ESCC, which we have previously shown to stimulate pre-metastatic niche formation, had an impact on the development of cancer metastases [15]. We first injected exosomes purified from ECA109 or KYSE410 cells grown in hypoxic environment, normoxic atmosphere, or PBS into the tail vein of nude mice. Following this, bioluminescent imaging was used to track the spread of the tumor after implanting luciferase labeled ECA109 and KYSE410 cells into the tail vein (Fig. 4A).

All mice were sacrificed 33 days later, and the tumor nodules that had developed on the surfaces of the lungs were recorded. In comparison to the control group, mice treated Norm-Exo or Hypo-Exo had more lung metastases. Mice from the Hypo-Exo group had more lung tumor nodules than those from the Norm-Exo group. In addition, animals treated with Hypo-anti-ZNF609-Exo had significantly fewer metastatic colonies in their lungs than mice treated with Hypo-Exo. Circulating exosomes from ESCC dramatically boosted metastases in distant organs, which is consistent with their impact on damaging endothelial barriers and promoting angiogenesis. (Figs. 4B, C)

### Circ-ZNF609 promotes tumor growth via enhancing angiogenesis and aggravates vascular endothelial cell dysfunction in ESCC

**Fig. 5:**
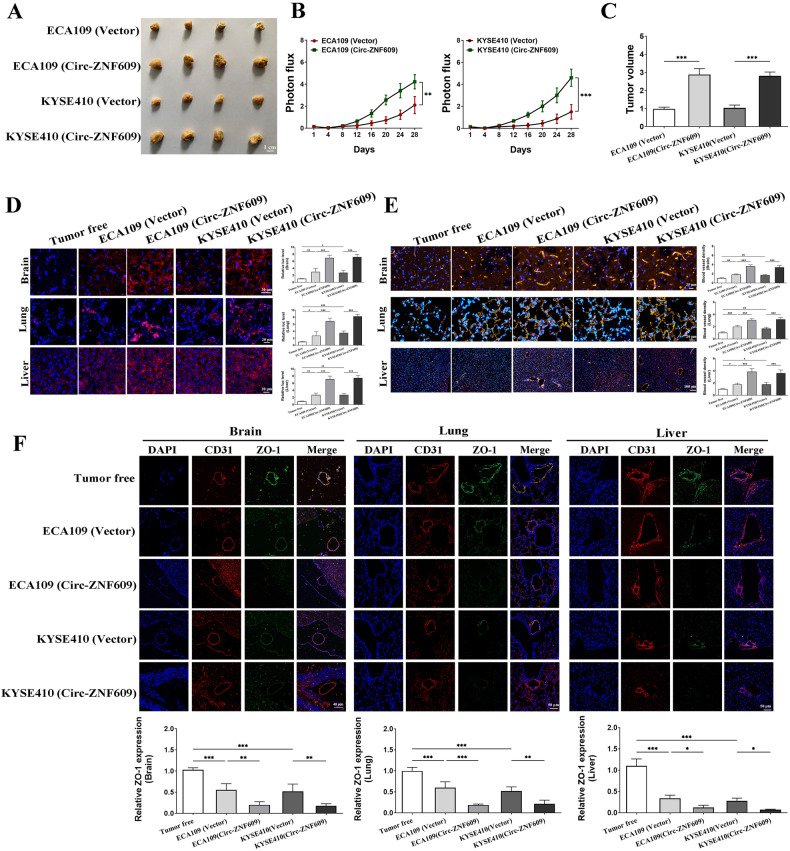
Circ-ZNF609 up-regulation facilitates angiogenesis and enhances vascular permeability in situ which results in ESCC progression. Xenograft transplanted tumor models were established through subcutaneously injecting ESCC cells (stably transfected with Circ-ZNF609 high expression vector or not). Tumor size was measured every four days. 28 days later, tumors were collected **A** and their volumes were measured **C**. Photon flux curves of luciferase capture are shown **B**. **D** Rhodaminedextran was administrated intravenously 3 h before sacrifice. Tumors were collected for fluorescence analysis to determine the vascular permeability. **E** Vascular density was evaluated by CD31 staining. **F** Tight junctions between HUVECs were determined by ZO-1 staining. (*p < 0.05; **p < 0.01; ***p < 0.001).

A mouse xenograft tumor bearing model was developed to explore the carcinogenic consequences of Circ-ZNF609 in vivo. Circ-ZNF609 was initially stably transfected into ECA109 or KYSE410 cells. Before the 28th day of sacrifice, bioluminescent imaging was used to gauge the tumor’s development. Circ-ZNF609 over-expression consistently sped up the development of ESCC (Figs. 5A–C). On the other hand, angiogenesis and vascular dysfunction were clearly impacted by tumor development. Importantly, Circ-ZNF609 triggered angiogenesis (Fig. 5E) and vascular leakiness (Fig. 5D) in distant tissues such the liver, lung, and brain. In line with the in vitro findings, the ZO-1 expression levels in each group were also assessed. The result revealed that Circ-ZNF609 up-regulation dramatically reduced the tight connection between endothelial cells (Fig. 5F). These findings highlight Circ-ZNF609’s crucial contribution to the promotion of ESCC.

### Circ-ZNF609 functions as a miR‑150‑5p sponge and interacts with HuR in HUVECs

**Fig. 6:**
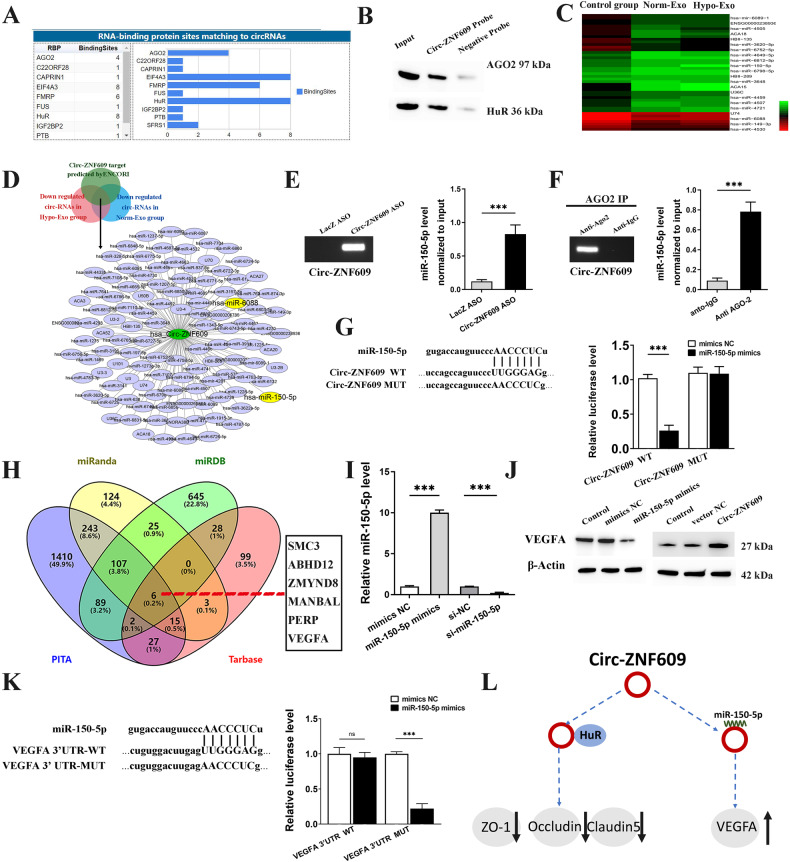
Circ-ZNF609 functions as a miR-150-5p sponge in HUVECs. **A** The potential binding sites of Circ-ZNF609 predicted by CircInteractome online database. **B** RNA pull-down assay was used to determine the interaction between Circ-ZNF609 and HuR or AGO2 in HUVECs. **C** HUVECs were incubated with Hypo-Exo from KYSE410, Norm-Exo from KYSE410 or without exosomes (control group) and then their miRNA expressions profile was analyzed. Heat map of the significantly down-regulated miRNAs in both Norm-Exo and Hypo-Exo group compared with control. **D** Construction of ceRNA network of Circ-ZNF09 predicted by ENCORI. **E** To verify that miR-150-5p binds to Circ-ZNF609, an ASO pulldown experiment was performed. **F** RIP assay was used to test the interaction of miR-150-5p with rabbit AGO2 and IgG antibodies in HUVECs. **G** Schematic diagram of Circ-ZNF09 luciferase reporter vectors carrying wild‑type (Wt) or mutant (Mut) miR‑150-5p binding sites (Left). Luciferase reporter assay to analyze the effects of miR‑150-5p on the luciferase activity of Circ-ZNF609 (Right). **H** Potential target mRNAs of miR-150-5p were predicted using miRnda, miRDB, PITA, and Tarbase. **I** The expression level of miR-150-5p in HUVECs after transfection. **J** VEGFA expression in HUVECs transfected with miR-150-5p mimics and Circ-ZNF609 overexpression plasmid. **K** Schematic diagram of VEGFA luciferase reporter vectors carrying wild‑type (Wt) or mutant (Mut) miR‑150-5p binding sites (left). Luciferase activity in HUVECs co-transfected with Luc- Circ-ZNF609 wild-type or mutant sequence and miR-150-5p mimics (right). **L** Regulation mechanism of Circ-ZNF609 on VEGFA through miR-150-5p and permeabilization related protein through HuR. (*p < 0.05; **p < 0.01; ***p < 0.001).

Circ-ZNF609 is frequently discovered in the cytoplasm of human cells, including myoblasts [16], rhabdomyosarcoma [17], and cholangiocarcinoma [18]. It primarily serves as a posttranscriptional regulator by functioning as a molecular sponge of target microRNA [19]. Based on the prior data, we hypothesized that Circ-ZNF609 may function as a ceRNA by actively sponging miRNAs. We found the probable interaction between Circ-ZNF609 and AGO2 (Argonaute 2), HuR (human antigen R) by looking through the CircInteractome online database, which forecasts the RNA-binding protein sites matching flanking sections of circRNA [20] (Fig. 6A). The RNA pull down test was used to confirm if AGO2 and HuR were enriched in micro-ribonucleoprotein complexes that contained Circ-ZNF609. We used a synthetic Circ-ZNF609 probe to incubate HUVEC cytoplasmic lysates, and then we used Western blot analysis to look for direct interactions between Circ-ZNF609 and Ago2 and HuR in the pull-down material. The possible binding of Circ-ZNF609 with AGO2 and HuR was validated by the results in Fig. 6B.

In order to find possible differentially expressed miRNAs in the control group, Norm-Exo, and Hypo-Exo groups of HUVECs, we next carried out miRNA screening. The complete list of genes with differential expressions was included in the supporting information and Figure S1A. As a result, the heat map for those miRNAs that showed the down-regulation in both the Norm-Exo and Hypo-Exo groups was chosen, and it is shown in Fig. 6C. Then, ENCORI projected the Circ-ZNF609’s possible target miRNAs (Fig. 6D). Two miRNAs, miR-6088 and miR-150-5p, were chosen for further validation in accordance with the overlap between internal miRNA screening findings and ENCORI predict results (Fig. 6D). In order to confirm that miR-150-5p and Circ-ZNF609 interact, an ASO pull down test was carried out in HUVECs. Figure 6E’s findings suggest that the Circ-ZNF609 designed ASOs functioned well. RT-qPCR analysis of the miR-150-5p level in the pull-down complex revealed a considerably enriched miR-150-5p level in Circ-ZNF609 binding complexes. MiR-6088, however, showed no change across the various groups (Data not shown).

To confirm the connection between miR-150-5p and Ago2, we performed an immunoprecipitation assay, and found that miR-150-5p included many Ago2 pull down ribonucleoprotein complexes (Fig. 6F). In addition, we performed a dual-luciferase reporter assay using plasmids containing both the wild-type and mutant forms of the Circ-ZNF609 gene. The findings indicated that miR-150-5p directly binds to Circ-ZNF609 and significantly decreases the quantity of luciferase in HUVECs with high expression of miR-150-5p (Fig. 6G). These findings point to the possibility that Circ-ZNF609 functions as a ceRNA sponge for miR-150-5p in HUVECs.

### Circ-ZNF609 acts as a ceRNA to coordinate VEGFA expression in HUVECs

We analyzed Kyoto Encyclopedia of Genes Genomes (KEGG) signaling pathways and Gene Ontology (GO) biological process enrichment for the target genes of miR-150-5p to better clarify the role of Circ-ZNF609 through miR-150-5p. The GO keywords angiogenesis, endothelial cell movement, cell adhesion, and cell cycle were considerably enriched in these genes, as shown in Figure S1B. Additionally, KEGG signaling pathways research revealed that target mRNAs for miR-150-5p were abundant in the Wnt signaling pathway and focal adhesion, among other signaling pathways.

Potential axis of Circ-ZNF609-miR-150-5p-target protein was investigated to learn more about the processes and underlying effects of miR-150-5p in HUVECs. The probable downstream targets of miR-150-5p were gathered by locating the junction of the miRanda, miRDB, PITA, and Tarbase (Fig. 6H). Western blotting study demonstrated that Circ-ZNF609 stimulated VEGFA protein synthesis, but overexpression of miR-150-5p significantly decreased VEGFA protein levels (Figs. 6I, J). Dual-luciferase reporter tests further demonstrated that miR-150 binds to the 3’-UTR of VEGFA (Fig. 6K). Because of this, we reasoned that VEGFA is a transcript that miR-150-5p can target (Fig. 6L).

### Exosomal Circ-ZNF609 regulates angiogenesis via miR-150-5p/VEGFA axis

**Fig. 7:**
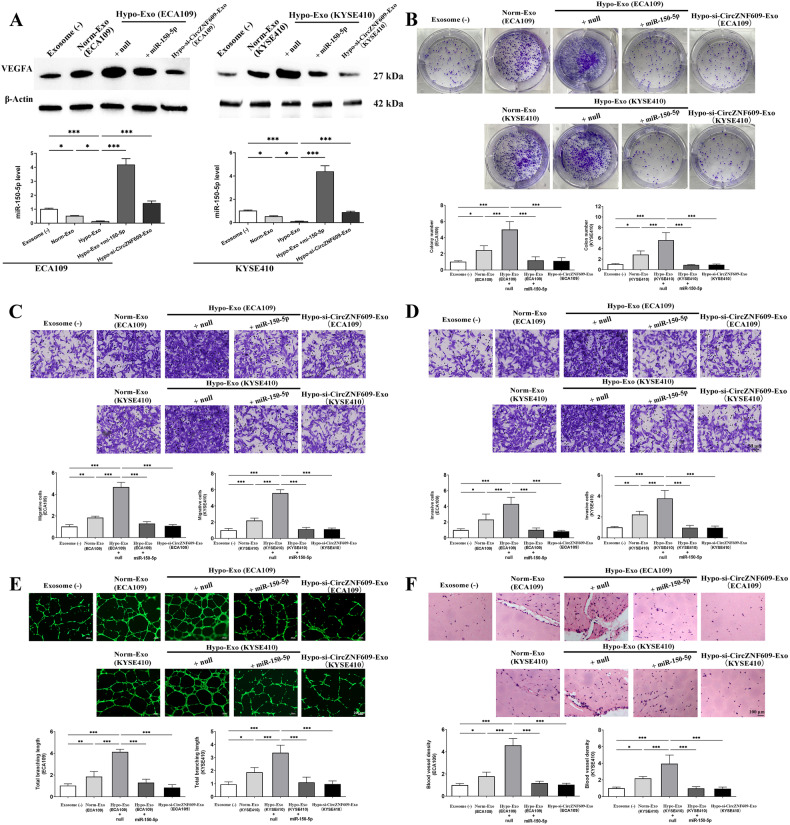
Hypoxia-induced exosomal Circ-ZNF609 from ESCC enhances angiogenesis and vascular permeability in vitro and vivo. **A** HUVECs were cultured with exosomes from ESCC cells that incubated in normoxic environment (Norm-Exo), hypoxic environment (Hypo-Exo + null), or Hypo-Exo loaded with Circ-ZNF609 inhibitor (Hypo-si-ZNF609-Exo). For Hypo-Exo Hypo-Exo+miR-150-5p group, HUVECs were transfected with miR-150-5p mimics and co-cultured with Hypo-Exo at the same time. Western blot results of VEGFA protein level (up) and RT-PCR results of miR-150-5p level (down) in HUVECs. The proliferation of HUVECs was detected by colony formation assay **B** Transwell assays were used to investigate the migratory **C** and invasive **D** abilities of HUVECs. **E** HUVECs were cultured with exosomes or not and then plated on matrigel. Representative pictures of tube formation were taken after stained with Calcein-AM. The tube formation ability was quantified by measuring the total branching length. **F** Matrigel containing exosomes, or not, were injected subcutaneously into the nude mice. In vivo neovascularization induced by exosomes was measured by H&E staining. Representative pictures of neovascularization were shown and quantified for blood vessel density. (*p < 0.05; **p < 0.01; ***p < 0.001).

Using RT-PCR and western blot analysis, we investigated into the miR-150-5p level and the amount of VEGFA protein in the HUVECs that consumed exosomes from the ESCC. We demonstrated that exosomes from ESCC enhanced VEGFA protein level and reduced miR-150-5p expression, while Circ-ZNF609 silencing in exosomes and restoration of miR-150-5p in HUVECs completely reversed the effects (Fig. 7A).

The colony formation and cell migration assays that were performed afterwards shown that both the Norm-Exo and Hypo-Exo treatments greatly increased in vitro cell proliferation (Fig. 7B) and migration (Fig. 7C, D). On the other hand, HUVECs stimulated by exosomes exhibited a more intricate network formation in vitro and in vivo. (Fig. 7E, F). Additionally, miR-150-5p high expression in HUVECs and Circ-ZNF609 depression in exosomes from ESCC suppressed the angiogenesis effects of Hypo-Exo.

### Exosomal Circ-ZNF609 represses translation of ZO-1, Claudin-1 and Occludin, and destroys vascular endothelial barriers by interacting with HuR

We investigated the possibility that Circ-ZNF609 inhibits ZO-1, Claudin-1, and Occludin translation by affecting HuR association with the mRNAs since HuR was found to directly regulate the translation of ZO-1, Claudin-1, and Occludin protein via interacting with their mRNAs [21, 22].

**Fig. 8:**
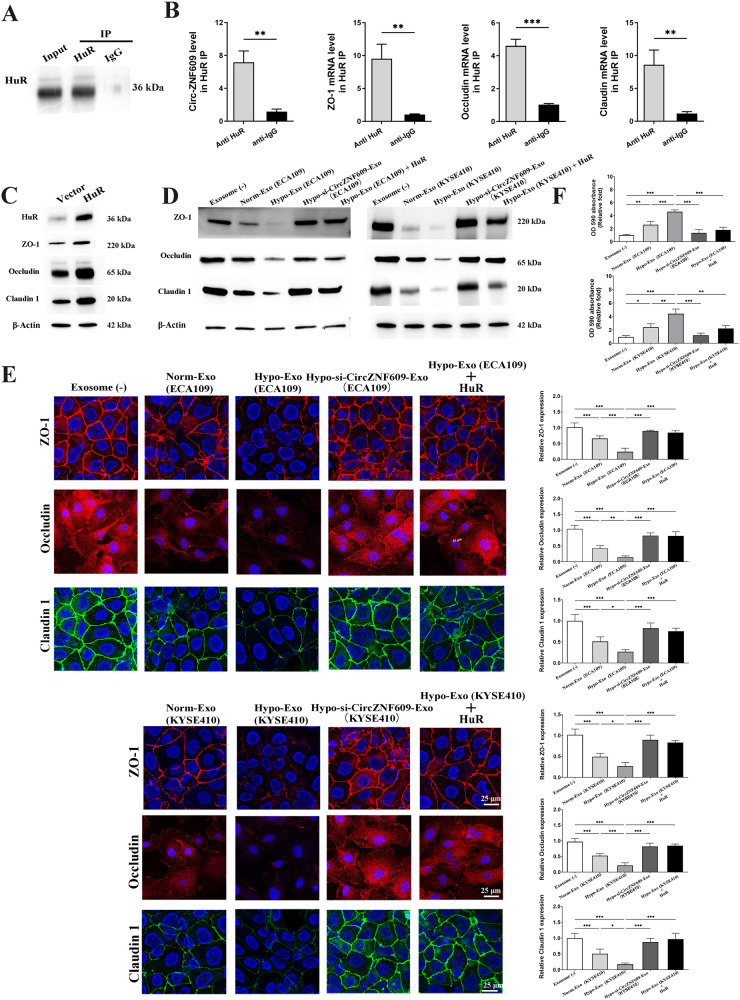
Exosomal Circ-ZNF609 inhibits translation of ZO-1, Claudin-1 and Occludin via interaction with HuR. Association of endogenous HuR with endogenous Circ-ZNF609 in HUVECs was measured by RIP assays. After cytoplasmic lysates were incubated with anti-HuR antibody or control IgG, RNA-protein complexes from cell lysates were isolated and measured by PCR analysis. The immunoprecipitation efficiency was examined by western blot **A** and the level of Circ-ZNF609, ZO-1, Occludin and Claudin-1 mRNAs in pull-down materials was checked by PCR **B** and western blot **C.** HUVECs were cultured with exosomes that incubated in normoxic environment (Norm-Exo), hypoxic environment (Hypo-Exo), or Hypo-Exo loaded with Circ-ZNF609 inhibitor (Hypo-si-ZNF609-Exo). HUVECs were treated with exosomes isolated from hypoxic cultured ESCC cells (Hypo-Exo), normoxic cultured- ESCC cells (Norm-Exo) or Hypo-Exo loaded with Circ-ZNF609 siRNA (Hypo-si-CircZNF609-Exo). For Hypo-Exo+HuR group, HUVECs were transfected with HuR over-expression plasmid and co-cultured with Hypo-Exo at the same time. Protein level of ZO-1, Occludin and Claudin-1 was analyzed with Western blot **D** and immunofluorescence **E**. **F** In vitro permeability assay was applied to determine the barrier function of endothelial cells after transfection.

Using anti-HuR and control IgG antibodies, we first conducted RIP experiments. Next, we isolated bound RNA in both RIP reactions (Fig. 8A). In order to compare HuR IP complexes to IgG IP, the enrichment of mRNA Circ-ZNF609, ZO-1, Occludin, and Claudin-1mRNA was evaluated. The data in Fig. 8B demonstrated a direct interaction between HuR and the mRNAs for Circ-ZNF609, ZO-1, Occludin, and Claudin-1. HuR was also produced ectopically in HUVECs, which raised the levels of ZO-1, Occludin, and Claudin-1 (Fig. 8C). It’s interesting to note that exosomes from ESCC, particularly Hypo-Exo, significantly decreased the amounts of ZO-1, Occludin, and Claudin-1 protein. However, these effects were reversed by HuR over-expression in HUVECs and Circ-ZNF609 knockdown in exosomes. (Figs. 8D, E).

Finally, we evaluated whether Circ-ZNF609 and HuR interacted to control in vitro epithelial barrier function. Exosomes from ECA109 and KYSE410, as expected, damaged the HUVECs’ barrier function and increased their permeability by increasing the flow of Rhodamine B-dextran between cells. HuR overexpression in HUVECs and Circ-ZNF609 knockdown in exosomes, however, mitigated the damaging impact of Hypo-Exo on epithelial barrier function in rescue tests (Fig. 8F). These results clearly support the hypothesis that elevated Circ-ZNF609 impairs epithelial barrier function by blocking HuR interaction with ZO-1, Occludin, and Claudin-1 mRNAs and thus suppressing their expression.

## Discussion

Metastasis has been considered the primary cause of elevated cancer mortality for decades [23]. During metastasis, tumor cells leave their primary sites, diffuse into the bloodstream, and colonize distant organs [24]. Only a limited percentage of circulating tumor cells are able to colonize and form metastases [25, 26]. According to the well-known “seed and soil” hypothesis, “seeds” (tumor cells) have a propensity to settle and germinate on “soil” (target organs) with specific micro-environmental conditions. Importantly, the organs in which metastasis formation occurs are not chosen at random and there is a discernible tendency. Bone, liver, brain, and lung are the most common metastasis organs [24].

It has been shown, in fact, that a pre-metastatic niche exists, with the formation of the target organ’s microenvironment occurring prior to tumor cell metastasis. [27]. The pre-metastasis niche provides a favorable environment for the spread of cancer cells. Subsequently, implanted cancer cells enter a proliferative state and result in the formation of a metastasis node [28]. Consequently, research into the mechanism of pre-metastasis niche formation contributes to the comprehension and treatment of cancer metastasis.

Exosomes derived from tumors regulate the recruitment and reprogramming of the tumor microenvironment due to their essential cargo of copious proteins and nucleic acid. During this process, the tumor microenvironment contains multiple positive feedback loops that contribute to the formation of pro-tumorigenic soil [8, 29, 30]. In our previous investigation, we demonstrated that exosomes from cancer cells promoted angiogenesis in the microenvironment of ESCC [15]. Here, we investigated whether these exosomes influence the formation of metastatic niches by regulating blood vessel permeability. Moreover, hypoxia and ischemia, which are common conditions in the microenvironment of tumors, have been widely recognized as key unfavorable factors for the rapid proliferation of tumors and as major stressors for the progression of cancer [31, 32]. To adapt to this adverse microenvironment, tumor cells must reshape their surrounding microenvironment not only for optimal survival and growth, but also to promote invasion and dissemination [33]. Consequently, we isolated exosomes from ESCC cells grown in hypoxia and assessed their function in promoting angiogenesis and degrading vascular endothelial barriers. Exosomes from ESCC were found to promote vascular formation and increase vascular permeability. In addition, exosomes for ESCC grown in a hypoxic environment (Hypo-Exo) performed better than those grown in a normoxic environment (Norm -Exo).

Circular RNAs are essential endogenous regulators for multiple biological processes as miRNA sponges, according to accumulating evidence. Our previous research suggested that hypoxia promoted ESCC tumor growth, vascular formation, and distant metastasis by modifying the circular RNA profile of ESCC exosomes [15]. Here, we identified Circ-ZNF609, a circular RNA that is transferable between cancer cells and stromal cells. Hypoxia treatment upregulated the exosomal Circ-ZNF609 expression from ESCC cells, which can be communicated into HUVECs via exosomes, and attenuated the tight junction between HUVECs, as indicated by the data. Through bio-information analysis and immunoprecipitation, we also identified the Circ-ZNF609/miR-150-5p/VEGFA and Circ-ZNF609/HuR/ ZO-1 axes.

In normal circumstances, VEGFA is a nutritive factor for vascular endothelial cells and a potent angiogenesis promoter that controls the stability of vascular tone [34]. VEGFA, however, perform essential biological regulatory functions in numerous disease models [35]. For example, it promotes angiogenesis in tumors, psoriatic lesions, and retinal vascular diseases [36–38]. VEGFA activates vascular regulatory-related transcription factors in endothelial cells, such as MAPK, phospholipase, protein kinase C, PI3K-AKT, and endothelial nitric oxide synthase, according to these studies [39–42].

In contrast, HuR is a powerful biological enhancer of epithelial barrier function, and its binding to target mRNAs is tightly regulated. [22, 43] Here, we showed that exosomal-Circ-ZNF609 from ESCC effectively disrupts of blood vessel barrier and weakened the adherent junction molecules at least partially by preventing HuR association with Claudin-1, Occludin and ZO-1 mRNAs, thereby inhibiting their expression in brain, lung and liver. The vascular endothelial barriers were also destroyed by the exosomes which assisted pre-metastatic niche formation and promoted remote metastasis. Moreover, Circ-ZNF609 was demonstrated to act as the sponge of miR-150-5p which could target VEGFA. In view of these published articles along with our data, we hypothesized that hypoxia might alter the exosomal Circ-ZNF609 expression from ESCC cells and sponges miR-150-5p which triggered VEGFA dysregulation and alter right junction protein expression by interacting with HuR in host cells-HUVECs.

To further investigate the effect of Circ-ZNF609, we also created a nude mouse model with a primary tumor. High Circ-ZNF609 expression increased vascular permeability and angiogenesis, which promoted the progression of ESCC. These findings revealed that elevated levels of exosomal Circ-ZNF609 induced angiogenesis and vascular leakage at both the primary tumor site and distant metastases. In fact, it has been demonstrated that the down regulation or loss of tight junction proteins is associated with the progression of cancer via alterations in cell migration and polarization [44–47]. In ESCC, decreased Occludin expression is associated with a high grade of histology and a poor prognosis [48]. Claudin was an independent histo-pathological risk factor for distal ESCC lymph node metastasis [49]. Both Occludin and ZO-1 exhibited decreased expression in undifferentiated adenocarcinomas of the human digestive tract [50]. Exosomes from ESCC, particularly hypoxic exosomes, downregulated the transcription level of tight junction protein in HUVECs and promoted angiogenesis, according to new evidence.

**Fig. 9:**
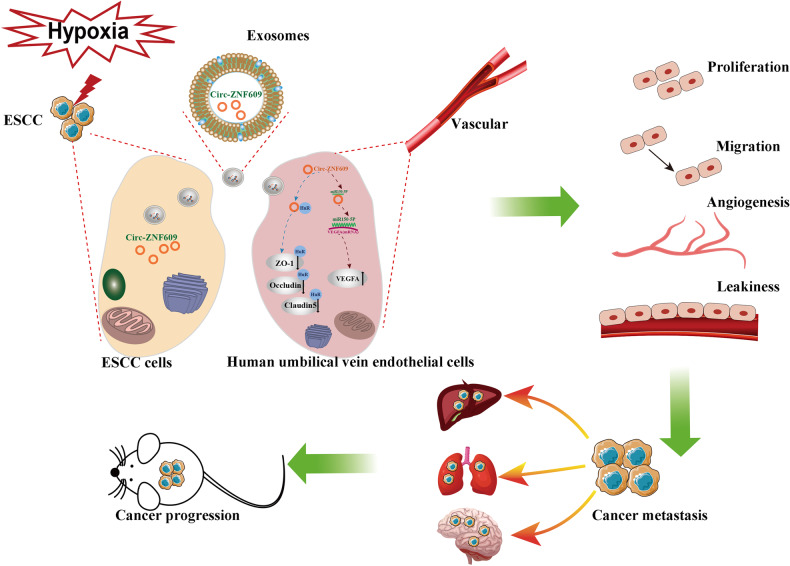
Schematic illustration. The molecular mechanism of hypoxia induced exosomal Circ-ZNF609 promotes esophageal squamous cell carcinoma pre-metastatic niche formation and metastasis.

On the one hand, damaged vascular endothelial boundaries and increased vascular density might aid cancer cell dissemination and proliferation at distal sites via multiple mechanisms. Initially, increased vascular permeability results in plasma protein leakage, which causes cell entrapment and tumor cell concentration. Second, an increase in the dispersion of tumor cells to distal sites activates autocrine-related signaling pathways that overpower other inhibitory pathways. Thirdly, additional circulating exosomes that infiltrate into secondary organs induce local physiology of cells into a pro-metastatic/tumor-supportive phenotype [6]. All of these variables contributed to the establishment of pre-metastatic niches and increased the number of tumor nodules in remote organs. However, exosomal Circ-ZNF609, particularly in hypoxic exosomes, enhanced vascular density, which alleviated hypoxia at the local site and fostered tumor growth. (Fig. 9).

In conclusion, our findings indicate that exosomal Circ-ZNF609 from ESCC cells plays a significant role in promoting tumor growth, metastatic niche formation, and distant metastasis. Our research sheds light on the potential future use of exosomes as a valued target for therapy to patients with ESCC.

## Materials and methods

### Cells and cell culture

Exosomes were excluded from fetal bovine serum (FBS) by ultracentrifugation at 100,000 g overnight (Beckman, USA), as previously depicted [51].

ECA109 and KYSE410 cells (verified by STR profiling) were cultured in RPMI 1640 (Gibco, New Zealand) with 10% FBS devoid of extracellular vesicles and 1% penicillin/streptomycin (Gibco). HUVECs (ATCC, PCS-100-010) have been cultured in ECM without exosomes (Science cell, USA). Humidified 37 °C incubators were used to cultivate all cell types with 5% carbon dioxide for normoxia and 5% carbon dioxide and 0.5% oxygen for hypoxia [15].

### Isolation and characterization of exosomes

Exosomes have been obtained from the culture medium of ESCC cells grown under normoxic or hypoxic conditions through differential centrifugation. First, conditioned media was centrifuged at 300 g for 5 min followed by 16,800 g for 30 min. After centrifuging at 100,000 g for 2 h at 4 °C, the supernatants were filtered through a 0.22 µm filter (Millipore). PBS was added to the exosome particles and they were centrifuged again for two hours at 100,000 g and 4 °C. Exosomes were fixed with paraformaldehyde and glutaraldehyde for electron microscopy. Before being examined with a Zeiss transmission electron microscope (Germany), the specimen was placed on a carbon-coated copper grid and stained with phosphotungstic acid. Nanoparticle Tracking Analysis System (Malvern, UK) was used to identify the size distribution and quantification of exosomes.

### Internalization of exosomes assay

PKH26 membrane dye (PKH26GL, Sigma-Aldrich, Germany) was utilized to fluorescently label exosomes for labeling purposes. The labeled exosomes were collected by ultracentrifugation after being rinsed with PBS. For the exosome internalization assay, labeled exosomes were mixed for 24 h with recipient cells. The cell skeleton of HUVECs was stained with PhalloidiniFluor 488 Reagent (Abcam, UK) and the nucleus was stained with DAPI (Solarbio, USA). Confocal microscopy pictures were taken using a Zeiss (Germany) laser scanning microscope. Cells (2 × 10^5^) were subject to subsequent experiments after stimulation with 1 × 10^9^ exosomes for 48 h.

### RNA silencing and plasmids

Shanghai GenePharma synthesized the miR-150-5p inhibitor (si-miR-150-5p), miR-150-5p mimics, and their control (inhibitor control/mimics control). To generate Hypo-si-Circ-ZNF609-Exo, Exo-FectTM Exosome Transfection Kit (System Biosciences) was used to load Circ-ZNF609 siRNA into exosomes. To generate with stable overexpressing Circ-ZNF609, Circ-ZNF609 cDNA was firstly synthesized and cloned into the pLC5-ciR vector (Geneseed) with Gibson Assembly Kit (New England Biolabs) and confirmed by sequencing. An empty vector served as negative control. The overexpression plasmid was then packaged with a Lentivirus-Packaging kit in HEK293T. ESCC cells were subsequently co-cultured with HEK293T supernatants and selected for sustained transfection.

### Western blotting

The BCATM Protein Assay Kit (Pierce, USA) and RIPA buffer (Roche) were used to extract lysates from cells and determine their protein concentrations. The proteins were then separated by SDS-PAGE and transferred to PVDF membranes (Millipore). Membranes underwent primary antibodies treatment with at 4 °C for 12 h afterward. Secondary antibody (Icllab, USA) incubation was completed by visualization of protein bands using chemiluminescence reagents (Millipore, MA, USA) and evaluation using Image J Software (National Institutes of Health). Primary antibodies included CD9 (Abcam, ab236630), TSG101(Abcam, ab30871), CD63 (Abcam, ab271286), Argonaute-2 (Abcam, ab186733), HuR (Abcam, ab200342), VEGFA (Abcam, ab46154), Angiotensin II (Novus), β-Actin (Abcam, ab8226), ZO-1 (CST, 5406), Occludin (CST, 91131) and Claudin 1(CST, 13255).

### RT-PCR

Trizol reagent (Invitrogen) was used to extract total RNA from cells, and the RevertTra Ace kit (TOYOBO) was used to reverse-transcribe the RNA in compliance with the manufacturer’s instructions. SYBR Green Master Mix (TOYOBO) was used for real-time PCR using an Applied Biosystems 7500 Fast Real-Time RCR System (Applied Biosystems). The miR-150-5p RT-PCR was performed using MystiCq® microRNA qPCR Assay Primer for has-miR-150-5p (Sigma). The Circ-ZNF609 amplification primers were designed in accordance with Legnini et al. [16].

### In vitro permeability assay

HUVECs, mixed with exosomes or not, were cultivated to confluence on the upper well of the transwell inserts for the in vitro permeability assay. The top well was filled with Rhodamine B isothiocyanate–dextran (average molecular wheight ~70,000, Sigma). One hour later, the medium in bottom well was retrieved, and the permeability of the HUVEC monolayer was determined through determining the absorbance at 590 nm. Normalization of absorbance values to the Exosomes (-) group served as the reference standard [6, 12].

### Colony formation assay

500 cells were seeded into each well of a 6-well plate, and the cells were grown in media with or without exosomes as stated above. Two weeks later, the colonies were tinted with crystal violet and tallied [15].

### Invasion and migration assay

Transwell chambers (24 wells) (Merck, Germany) were used to implant HUVECs in the top compartment with or without Matrigel (BD Biosciences, USA), as originally presented. The membranes’ undersides have been colored with crystal violet and photographed two days later [15].

### Tube formation assay

A 96-well plate covered with Matrigel Basement Membrane Matrix (BD, United States) was either treated with exosomes or left untreated. Calcein-AM (Sigma-Aldrich, Germany) was used to stain the cells, and images were taken using a Zeiss fluorescence inverted microscope.

### Matrigel plug assay

BALB/c nude mice were administered subcutaneously in ventral region with Matrigel (BD Biosciences) (500 μl) combined with normoxic or hypoxic exosomes. After 7 days of vaccinations, the matrigel was removed and fixated [15].

### Immunohistochemistry

Tissues were formalin-fixed and paraffin-embedded. The sliced sections of matrigel plug were stained with hematoxylin and eosin. The tissue staining was observed using an inverted Zeiss microscope (Germany).

### Immunofluorescence

HUVECs were cultivated to confluence on glass slipcovers before their fixation with 4% paraformaldehyde for 10 min. Fluorescent labeling antibodies were applied to monolayer cells after blocking with 3% BSA for 30 min at room temperature. Afterwards, sections were incubated with DAPI (Solarbio, China), a nucleus-specific anti-fluorescence agent.

Tissues embedded in paraffin were sectioned and placed on glass transparencies. Sections were immersed in 3% H_2_O_2_ and serum albumin to inhibit endogenous peroxidase activity and nonspecific binding, respectively. After that, primary antibodies and secondary antibodies conjugated with Alexa Fluor 488 or 594 (Abcam, USA) were added, and the sections were incubated at 4 °C for 24 h. Anti-fluorescence quenching agent DAPI (Solarbio, China) was used to counterstitch nuclei. Image acquisition was performed using a Zeiss LSM510 Meta (Zeiss, Germany).

### RNA Immunoprecipitation (RIP)

Abcam was the vendor for the acquisition of both the Ago2 and HuR antibodies, as well as the negative control. Immunoprecipitation of RNA was carried out using the Immunoprecipitation reagent (ab206996, Abcam) per the manufacturer’s instructions. At first, antibody-coupled magnetic beads were left to incubate with lysed cells at 4 degrees Celsius for a whole night. The complexes of beads and antibodies were then thoroughly washed. Purified RNA was extracted using Trizol reagent (Invitrogen) per the producer’s instructions after proteinase K digestion.

### RNA pull-down

To conduct an RNA pull-down test, we utilized the Thermo Scientific Pierce Magnetic RNA-Protein Pull-Down Kit. The Circ-ZNF609 was labeled using the 3′ desthiobiotinylation kit from Thermo Scientific. After adding streptavidin magnetic beads, the reaction was left to incubate at room temperature. Place the tube on the magnetic stand to gather the beads. To collect the beads, position the tube on a magnetic stand. Conduct western blot analysis after eluting the RNA-binding protein complexes after rinsing.

### Tumor metastasis models construction

Male BALB/c nude mice (six weeks) were ordered from Charles River (Beijing, China). All procedures involving animal research have been examined and authorized by the Animal Investigation Committee of First Hospital of Qinhuangdao. We followed all applicable ethical guidelines for animal testing and research.

Exosomes were injected intravenously every other day for three weeks to simulate constant and broad stimulus by exosomes from ESCC cells in an experiment testing exosomes’ educational efficacy. A day after the last injection, 2 × 10^6^ luciferase-labeled ESCC cells administered intravenously to rodents. Then, D-Luciferin (Perkin Elmer) was administered intraperitoneally to rodents prior to bioluminescent imaging (IVIS Spectrum, PerkinElmer). The mice were allocated to experimental groups in a random manner, and no data were omitted. The investigators were aware of the group allocation during the procedure. Following the sacrifice after 33 days, tissues were preserved with 4% paraformaldehyde for additional analysis.

### Luciferase assay

PsiCHECK-2 vectors were built with either wild-type (WT) or mutant (MUT) miR-150-5p binding sites in the 3’UTR of human VEGFA (or Circ-ZNF609). 48 h after co-transfection of miR-150-5p or a negative control alongside both vectors into HUVECs, luciferase activity was measured.

Exosomes from ESCC cells were incubated with HUVEC cells for 48 h in a separate experiment. Using a Dual-Luciferase Reporter Assay System, we quantified luciferase activities and normalized Renilla luciferase activities to firefly luciferase expression to determine their relative strengths.

### In vivo nude mice xenograft model

Subcutaneous injections of luciferase-labeled ECA109 or KYSE410 cells (either persistently transfected with miR-150-5p enhanced expression vectors or not) were made into the hind limbs of rodents, as illustrated before. Then, D-luciferin (Perkin Elmer) was administered intravenously to rodents prior to bioluminescent imaging (IVIS Spectrum, PerkinElmer). After 28 days, tumors were collected, and the rodents were sacrificed.

### In vivo permeability assays

Exosomes were administered intravenously to rodents every two days for three weeks for generating continuous and systemic stimulation by exosomes from ESCC cells. One day following the final injection, nude rodents were sacrificed, and their tissues were fixated with 4% paraformaldehyde for immunofluorescence evaluation.

In contrast, rhodaminedextran (100 mg/kg) was injected intravenously into null rodents following pretreatment. Three hours later, mice underwent to a trans-cardial perfusion to remove excess dye. For fluorescent microscopy, imbed tissues with Tissue-Tek O.C.T. Compound (Sakura, CA) and stained with DAPI [6, 12].

### Co-expression network construction

Up- or down-regulated circular RNAs in both the Norm-Exo and Hypo-Exo groups were identified. Using ENCORI the partnership between miRNA and Circ-ZNF609 was predicted [52]. Through miEAA, the predicted target miRNAs were subjected to enrichment analysis and annotation [53]. Using Cytospace, the co-expression network was plotted.

### MicroRNA profile analysis of HUVECs internalized exosomes

To begin, HUVECs were separated into the three categories as illustrated before [15]. Following the collection of HUVECs, miRNAs were isolated and Beijing CNKINGBIO Biotechnology Company Limited analyzed microRNA profiles using Affymetrix miRNA 4.0, human (Affymetrix, USA).

Briefly, biotin-labeled poly-A tails were attached to the 3′-end of miRNA. After that, the GeneChip 3000 7 G was used to scan miRNA with an Affymetrix GeneChip miRNA Array 4.0. Array (Affymetrix). The scanner picks up fluorescence signals and processes them using GCOS software to get a signal value. Cluster 3.0 software and significance analysis of microarrays (SAM) were utilized to normalize the value, and miRNAs with fold change≥1.5 were deemed differentially expressed genes (DEGs).

### Statistical analysis

Using GraphPad Prism 7 software, statistical analyses were carried out. Each experiment was repeated a minimum of three times. All quantitative values were presented as the mean standard deviation. Using one-way ANOVA, followed by Holm-Sidak’s multiple comparison tests or an unpaired t-test, the differences among/between groups were analyzed. We considered P < 0.05 to be statistically significant. For figure presentation, Adobe Photoshop CS6 was used.

### Supplementary information

figure legend for supplementary figure

Supplementary Fig. 1

Supplemental Material- western blot

Significantly different expressed miRNAs

## Data Availability

The data supporting the findings of this study are available within the article and its supplementary materials.
