# Supplementary material for: Hypoxia induced exosomal Circ-ZNF609 promotes pre-metastatic niche formation and cancer progression via miR-150-5p/VEGFA and HuR/ZO-1 axes in esophageal squamous cell carcinoma
Source: Cell Death Discov. 2024 Mar 12;10:133. doi: 10.1038/s41420-024-01905-8 (PMC10933275; doi:10.1038/s41420-024-01905-8)
Supplement: Supplementary file 1 — figure legend for supplementary figure [file 41420_2024_1905_MOESM1_ESM.docx]

**Figure legend**

**Supplementary Figure 1.** A. Scatter-Plot of differentially expressed miRNAs of HUVECs in the control group and Norm-Exo group (left). Scatter-Plot of differentially expressed miRNAs of HUVECs in the control group and Hypo-Exo group (right). Dots above the top line (blue) and below the bottom line (red) indicated the fold change of the RNAs is more than 1.5 between the two groups. B. Relative miR-150-5p level in HUVECs treated with miR-150-5p mimics or si-miR-150-5p. C. GO biological process enrichment and KEGG pathway enrichment analysis of the target gene of miR-150-5p.
