# Supplementary figures and images for: Hypoxia induced exosomal Circ-ZNF609 promotes pre-metastatic niche formation and cancer progression via miR-150-5p/VEGFA and HuR/ZO-1 axes in esophageal squamous cell carcinoma
Source: Cell Death Discov. 2024 Mar 12;10:133. doi: 10.1038/s41420-024-01905-8 (PMC10933275; doi:10.1038/s41420-024-01905-8)

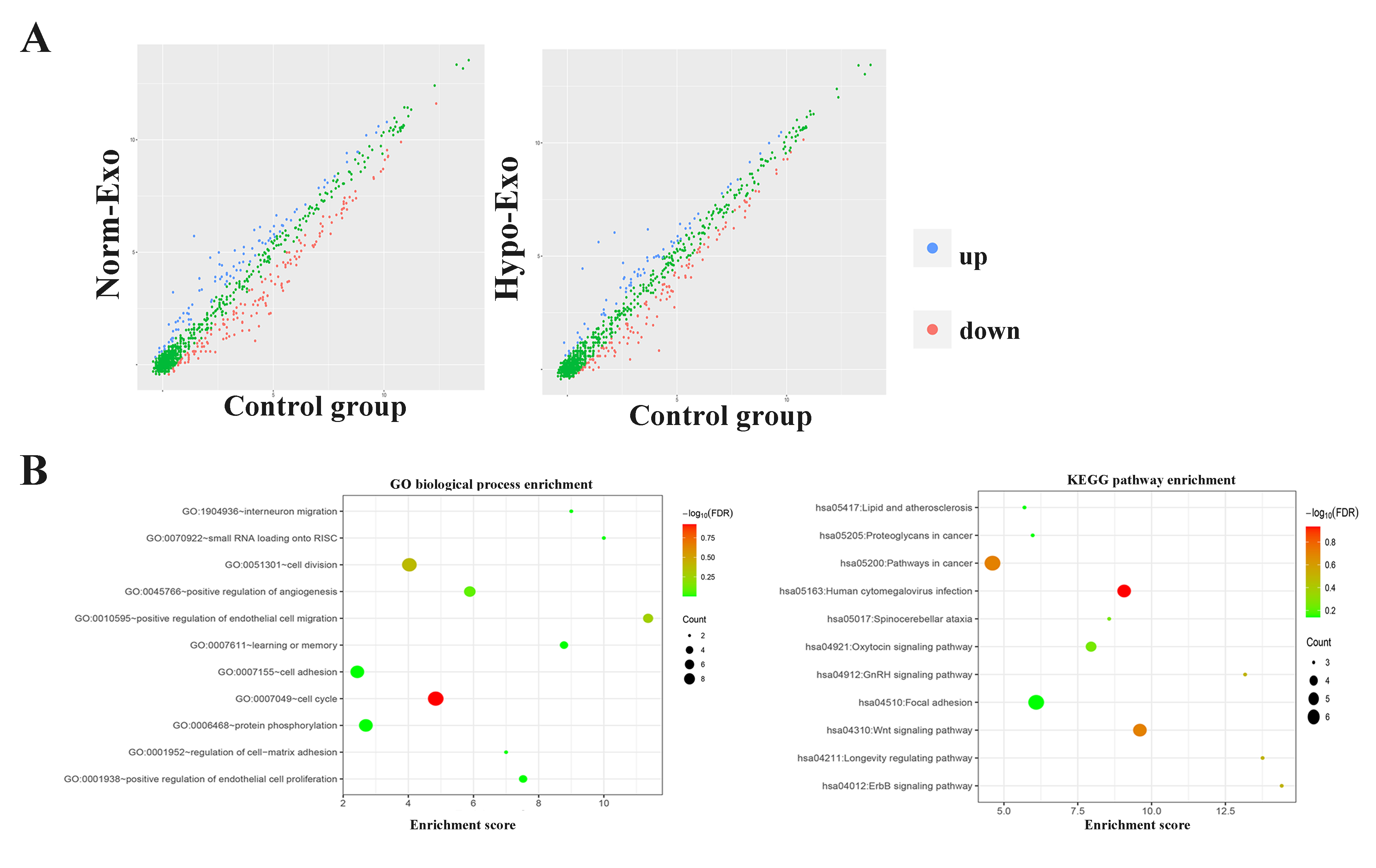

Supplement: Supplementary file 2 — Supplementary Fig. 1 [file 41420_2024_1905_MOESM2_ESM.jpg]

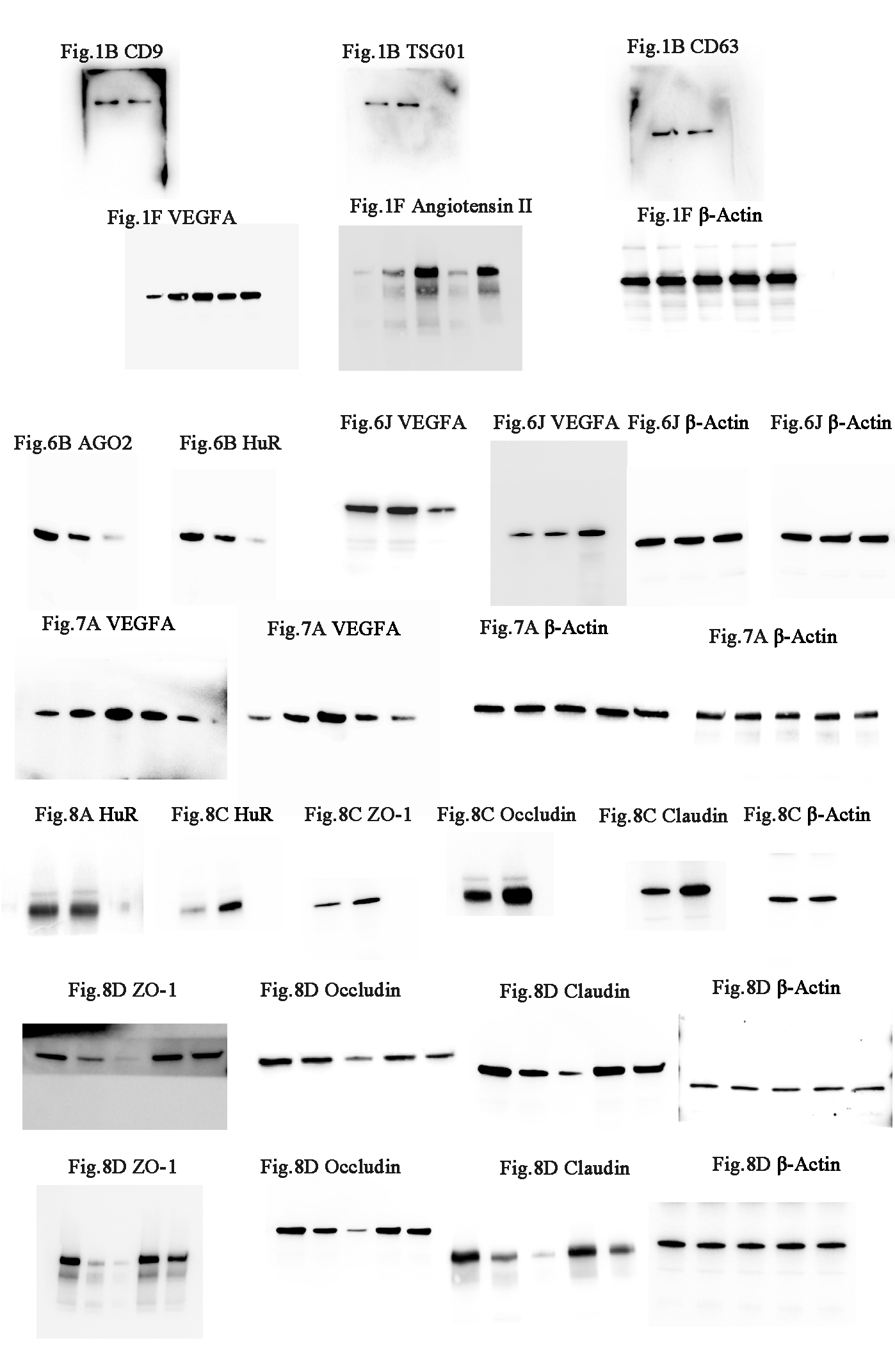

Supplement: Supplementary file 3 — Supplemental Material- western blot [file 41420_2024_1905_MOESM3_ESM.jpg]
